# Sugar-sweetened beverage/snack consumption and its determinants among infant and young children aged 6–23 months in twelve Sub-Saharan African countries: Evidence from 2019–2023 Demographic Health Survey data

**DOI:** 10.1371/journal.pone.0338308

**Published:** 2025-12-04

**Authors:** Birtukan Gizachew Ayal, Abebe Kassa Geto, Sefineh Fenta Feleke, Ali Yimer, Atitegeb Abera Kidie, Natnael Amare Tesfa, Esuyawkal Mislu, Molla Hailu, Hassen Ahmed Yesuf

**Affiliations:** 1 Department of Public Health, College of Health Science, Woldia University, Woldia, Ethiopia; 2 School of Medicine, College of Health Sciences, Woldia University, Woldia, Ethiopia; 3 Department of Midwifery, College of Health Sciences, Woldia University, Woldia, Ethiopia; 4 Department of Biomedical Sciences, School of Medicine, College of Health Sciences, Woldia University, Woldia, Ethiopia; Shandong University, CHINA

## Abstract

**Background:**

Sugar-sweetened beverages or snacks are limited in nutritional value. Excess consumption of sugar-sweetened beverages or snacks in early childhood is associated with inadequate micronutrient intake, being overweight or obese, and developing chronic diseases later in life. There is scarcity of information specific to sugar-sweetened beverages or snacks consumption prevalence and its determinants in Sub-Saharan Africa Countries. This study aimed to determine the pooled prevalence of sugar-sweetened beverage or snack consumption and its determinants among infants and young children aged 6–23 months.

**Methods:**

A cross-sectional study design was conducted using demographic and health survey data conducted from 2019 to 2023 from twelve Sub-Saharan African countries. A weighted sample of 23,145 children aged 6–23 months was included in the study. Multilevel mixed-effects logistic regression analysis was used to determine the factors associated with the dependent variable. The level of statistical significance was declared with a p-value < 0.05 and an adjusted odds ratio with a 95% confidence interval.

**Results:**

The pooled prevalence of Sugar-sweetened beverage or snack consumption was 25.40% (95% CI: 24.84% − 25.96%). In multilevel multivariable logistic regression analysis, children aged between 9−11(AOR = 1.95 95% CI: 1.62, 2.35), 12–17(AOR = 2.83; 95% CI: 2.26, 3.54), and 18−23 months (AOR = 3.77;95% CI: 3.07, 4.63), media exposure (AOR = 1.59; 95% CI:1.28, 1.98), children from households with middle (AOR = 1.39; 95% CI: 1.11, 1.73) and rich (AOR = 2.31; 95% CI: 1.87, 2.85) wealth status, post natal checkup (AOR = 1.18; 95%CI:1.05,1.33), maternal ANC visit (AOR = 1.60; 95% CI: 1.15, 2.22), and high community media exposure ((AOR = 2.22;95%CI:1.65,5.81) were positively associated significant factors whereas currently breast feeding children (AOR = 0.70; 95% CI: 0.59,0.82), older age at first birth (AOR = 0.88,95% CI: 0.81, 0.96), presences of more than one under-5 children in the household (AOR = 0.89, 95% CI: 0.80, 0.99), mothers don’t perceive distance to health facility as big problem (AOR = 0.86; 95% CI:0.76, 0.98), and children reside in rural (AOR = 0.83;95%CI:0.68,0.95) were negatively associated with sugar sweetened beverage or snack consumption.

**Conclusion:**

In this study, one out of four children consumed sugar-sweetened beverages or snacks. Current age of child, current breastfeeding status of child, media exposure, wealth index, maternal age at first birth, post natal checkup, maternal ANC visit, number of under-five children in the household, distance to health facility, place of residence, and community level media exposure were significant factors with sugar-sweetened beverage or snack consumption. Therefore, it is recommended to raise awareness about the health risks of sugar-sweetened beverage and snack consumption, enforce restrictions on their advertisement, strengthen nutrition-focused counseling within maternal and child health services with special attention for older age children, promote breastfeeding, and give special attention to challenges related to health facility accessibility, and support for young mothers.

## Introduction

Consuming sugar-sweetened beverages or snacks refers to the intake of any foods or liquids that contain added sugars, including items such as soda, energy drinks, sports drinks, teas, coffee, and fruit juices, as well as snacks like biscuits, chocolates, candies, and cake-like snacks such as cake-bars, pastries, muffins, and cookies [1]. Both sugar-sweetened beverages and snacks are limited in nutritional value and contribute no nutrients other than energy, and may displace more nutritious foods [1,2]. Excess consumption of sugar sweetened-beverages or snacks is associated with inadequate micronutrient intake and being overweight or obese and chronic diseases later in life [2].

Intake of sweet drinks or snacks frequently in early childhood increases the desire for sweetness, resulting in increased consumption and preference for sweet-tasting foods later in life. They contribute no nutrients other than energy and may displace more nutritious foods. As a result, excessive consumption of sweet beverages or snacks is also linked with poor dietary quality and health, such as inadequate micronutrient intake, lower intake of protein, low dietary fiber, being overweight or obese, poor growth outcomes, and developing chronic diseases later in life [2–5]. The introduction of sugar-sweetened beverages or snacks (SSB/SN) commonly takes place during a critical developmental stage when taste preferences are being formed, and these early-established preferences frequently persist into later childhood and adulthood [6]. It is evidenced that such introduction of SSB/SN during early life, typically before 12 months of age, are linked to overweight or obesity, the risk of type 2 diabetes, the development of dental caries, increasing fatty liver, the risk for cardiovascular disease, and some types of cancers [7–9].

A shift in dietary patterns and energy expenditure called nutrition transition, is a major concern worldwide and especially in low and middle-income countries, including those in sub-Saharan Africa (SSA) [10]. In those countries, dietary habits are shifting towards increased consumption of added sugars, unhealthy saturated fats, salt, and ultra-processed carbohydrate sources. Food items that are made commercially are often salt, sugar, saturated and/or trans fatty acids, and energy-dense but poor in essential nutrients [1]. Consequences of nutrition transition are more severe in African countries than in developed countries as they pose a double burden of under-nutrition and obesity to the community, house-holds and individuals [11]. Thus, in developing countries such as sub-Saharan Africa, greater efforts should be directed towards minimizing rapid dietary shifts towards consumption of high energy dense foods by children to mitigate possible development of chronic diseases in future life.

The period from conception to age two, is the most crucial for the development of their body, brain, metabolism, and immune system [12]. The ability of a child to develop, learn, and thrive is significantly impacted by how well or how poorly mothers and children are fed and cared for throughout this period. This is also the period when the foundations for their long-term health are set [1,12]. To improve growth, health and behavioral development, the World Health Organization (WHO) and the United Nations International Children’s Emergency Fund (UNICEF) developed the global strategies or guiding principles for infant and young child feeding (IYCF) practice for children under 2 years of age [1]. Such strategies and guidelines recommend to limiting or avoiding sugar-sweetened beverage or snacks for infants and young children [1].

Globally, an estimated of 35 million under five children were overweight or obese. Once considered a high-income country problem, overweight is on the rise in low- and middle-income countries including Africa. In Africa, the number of overweight children under 5 years has increased by nearly 12.1% since 2000 [13]. Those are linked to the increased consumption of SSB/SN and energy-dense, nutrient-poor snack foods among infants and young children [14].

In sub-Saharan Africa (SSA), the intake of SSB/SN is increasing and is mainly due to economic development, globalization, or urbanization which leads to more accessibility for such beverages or snacks [10]. Despite limited data on specific Sugar-sweetened beverage or snack consumption, previous studies showed that high unhealthy food consumption is a public health challenge in SSA due to poor maternal education, economic development, urbanization, and an increase in affordability or accessibility as well as market advertising [15,16].

Despite the WHO/UNICEF recommendation to limit or avoid sugar-sweetened beverages or snacks for infants and young children, there is scarcity or dearth of information specifically to sugar sweetened beverage or snack consumption prevalence and its determinants using the most recent DHS data in SSA by considering the recent IYCF guideline. The previous studies conducted mainly focused on the combination of all unhealthy feeding practices, and are from the old SSA DHS data set and only analysis from five SSA countries [15,16]. Therefore, this study aimed to determine the pooled prevalence of sugar -sweetened beverage or snack consumption and its determinants among infants and young children aged 6–23 months in twelve sub-Saharan African countries using the most recent(2019–2023) demographic and health survey data. Such findings will be used for designing evidence-based interventions to mitigate sugar sweetened beverage or snack consumption among infants and young children and developing policies promoting healthier dietary habits from earliest stages of life.

## Methods

### Study design, period, and setting

An analytical cross-sectional study was conducted to determine the pooled prevalence of SSB/SN consumption and associated factors using the most recent Demographic and Health Survey (DHS) data from twelve sub-Saharan African (SSA) countries. The DHS datasets were taken from studies that were conducted from 2019 to 2023. DHS from twelve sub-Saharan African countries including Burkina Faso (2021), Côte d’Ivoire (2021), Gabon (2019), Ghana(2022), Gambia(2019), Kenya (2022), Madagascar(2021), Mauritania(2019–21), Rwanda(2019–2020), Sierra Leone(2019), Senegal (2023), and Tanzania(2022) were used. The data were appended to figure out the pooled prevalence of SSB/SN consumption and its determinants among infant and young children aged 6–23 months in 12 sub-Saharan African countries.

### Data sources, sampling, and population

The DHS is a nationwide survey mostly collected every five years across LMICs. Different datasets, including those for children, males, women, births, and households are included in the survey for each country. The DHS surveys adhere to the same standard approach for sampling, questionnaires, data collection, and coding [17]. A stratified two-stage cluster sampling technique is used in the survey. The stages include development of a sampling frame, involving a list of sampling units(PSUs) or enumeration areas(EAs), which covers the entire country at the first stage and systematic sampling of households listed in each cluster or EA in the second stage [18,19]. In this study, the kid’s record (KR file) was used. The data is available from the DHS’s official website (https://dhsprogram.com/data/available-datasets.cfm). Since the data set is freely available from the measure DHS official database, anyone can access it through a reasonable request.

**Fig 1:**
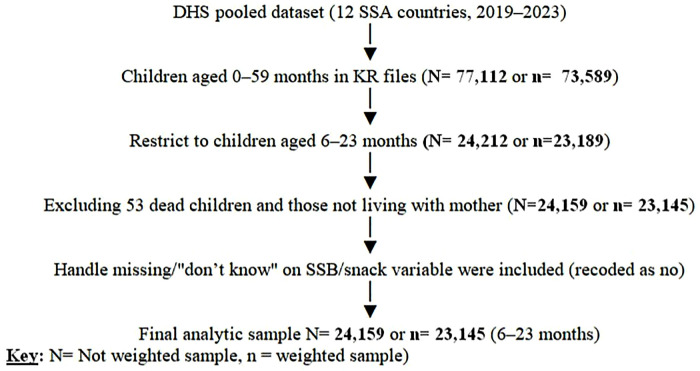
Flow diagram of analytic sample for SSB/SN consumption among children 6–23 months in SSA.

The source population included children aged 6–23 months lived with their mother over the five years before the survey period in Sub-Saharan Africa. This study covered all children aged 6–23 months in the five years preceding the survey in the selected enumeration areas (EAs) in each country. Children who have died are excluded from the study. According to the DHS recode guideline for management of missing values, “missing” and “don’t know” replies on whether the child was given sweet beverages or snacks yesterday throughout the day were included from the study but were regarded as not consuming sweet beverages or snacks [20]. Finally, a total weighted sample of 23,145 infant and young children aged 6–23 months was included in the final analysis (**Table 1 and Fig 1**).

**Table 1:** Weighted percentage and sample of Study subjects included by the country and year of survey in Sub-Saharan African countries, DHS from 2019- 2023(weighted n = 23,145).

Country	Survey year	Weighted sample(n)	Percentage (%)
Burkinfaso	2021	1,716	7.41
Côte d’Ivoire	2021	1,419	6.13
Gabon	2019	1,824	7.88
Ghana	2022	1,376	5.94
Gambia	2019	1,133	4.89
Kenya	2022	4,982	21.53
Madagascar	2021	1,866	8.06
Mauritania	2019−21	3,150	13.61
Rwanda	2019-2020	1,247	5.39
Sierra Leone	2019	1,458	6.30
Senegal	2023	1,370	5.92
Tanzania	2022	1,604	6.93
**Total weighted sample size**		**23,145**	

### Study variables

#### Outcome variable.

The outcome variable of this study was sugar sweetened beverage or snack consumption (coded as “0” didn’t consume sugar sweetened beverage or snack, and “1” for consumed sugar sweetened beverage or snack). Sugar sweetened beverage or snack consumption is defined as intake of any liquids or snacks that are sweetened with added sugars, such as soda, energy drinks, sports drinks, teas, coffee, fruit drinks, biscuits, chocolates, candies, cakes, and cake-type snacks such as pastries, cookies, etc [1] during the previous day. If any amount or type of such liquids or snacks has been taken, children are considered as “consumed sugar sweetened beverage or snack”.

#### Independent variables:

both individual and community level variables were considered by reviewing literature [15,16,21,22] and based on their availability in the DHS dataset.

**Individual level variables include:** Age of child in month, sex of child, current breast feeding status, preceding birth interval, birth order, birth size, twin status, initiation time of breast feeding after delivery, age of mother, maternal education, maternal occupation, maternal age at first birth, media exposure(reads newspapers/magazines, or listens to the radio, or watch television at least once a week), wealth index, family size, number of under-five children, sex of household head, distance to water source, Place of delivery, Post natal check (PNC), mode of delivery, ANC attendance, frequency of ANC, and timing of ANC.

**Community level variables include:** Place of residence (urban/rural), community ANC coverage (low/high), community media exposure (low/high), community level poverty (low/high), and community level literacy (low/high). These variables were categorized as low and high according to a median value of ≤ 50% and >50% respectively.

### Data management and analysis

Data extraction, recoding, cleaning, and analysis were done using Stata version 17 statistical software. To get a reliable estimate, manage sampling errors, and keep representativeness of the survey, the data were weighted using sampling weight (v005/1000000) before doing any statistical analysis. Variables included in this study were recoded into categorical and categorical variables were further re-categorized. Descriptive analysis was employed to present data in frequencies and percentages. The hierarchical nature of the DHS data, which violates the independent assumptions of the standard logistic regression model, was handled with a multilevel logistic regression analysis. Children in the same cluster are more likely to be similar to each other than children from other clusters, which indicates to take into account the cluster variability by using a multilevel logistic regression model. Therefore, multilevel mixed-effects logistic regression was used to identify the factors associated with SSB/SN consumption.

In multilevel mixed-effect logistic regression, the null model- a model with only outcome variable (SSB/SN consumption), Model I- a model with only individual-level variables, Model II- a model with only community level variables, and Model III(final model)- a model with both individual and community level variables were fitted.

Assessment of community level clustering, and model comparison were done. The Interclass Correlation Coefficient (ICC) and Median Odds Ratio (MOR) were checked to assess whether there was clustering or not. Model selection was guided by information criteria, including the Log-Likelihood Ratio (LLR) and Akaike Information Criterion (AIC). The model with the lowest AIC value (Model III or final model) was identified as the best-fitting model. Multicollinearity was checked using VIF which was within acceptable limit between 1–10. Bivariable multilevel logistic regression was conducted to select variables that will be entered in the multivariable multi-level logistic regression to adjust potential confounders. At the bivariable analysis, variables with a *p*-value ≤ 0.25 were considered for multivariable analysis. In the multivariable multilevel analysis, the Adjusted Odds Ratio (AOR) with 95% Confidence Interval (CI) was considered to declare the statistical significance of the association with SSB/SN consumption. Finally the results were presented using texts, graphs, and frequency tables.

### Ethical considerations

This study used publicly available secondary data from the DHS website. Permission was granted to download and use the data from (https://dhsprogram.com/data/available-datasets.cfm before conducting the study. Since the study was a secondary data analysis based on publicly accessible DHS records, participant participation and ethical approval were not required. There were no patients or members of the public involved since this study used a publicly available data set. Furthermore, as the study was based on secondary data analysis, gaining participants’ consent was not applicable. The procedures involved for DHS public use of datasets that do not allow respondents, or households to be identified.

## Results

### Socio-demographic characteristics of study participants

In this study, 23,145 weighted children aged 6–23 months were enrolled in Sub-Saharan African countries. The mean age of mothers was 28.45 ± 0.04 years, and approximately half, 10,809(46.70%) of respondents were in the age group between 25–34 years. About 8,786 (37.96%) mothers had secondary and above education level. Regarding marital status, the majority, 19,698 (85.11%) of mothers are currently married and above half, 13,309 (57.53%) of them had work. The majority, 10,116 (43.71%) of mothers in SSA countries had poor socioeconomic conditions. Nearly, three-fourth, 17,045 (73.64%) of mothers were exposed to at least one of the media sources (watching television, listening to the radio or reading a newspaper). More than one under five children were live in about 14,535 (62.80%) of respondents households.

The majority 19,799.65 (82.88%) of mothers in SSA countries, were delivered at health facilities, and only 7,779 (33.61%) of them had PNC checkups. About 21,294 (92.00%) mothers had at least one ANC visit during their pregnancy.

### Child characteristics

The mean age of children was 14.18 ± 0.034 months, and 8,129 (35.13%) of them fall in the age category of 12–17 months. More than three-fourths, 17,932 (77.48%) of the study subjects were currently breast feeding, and more than half 15,753 (68.06%) of them put in to breast milk immediately after delivery.

More than half 13,962 (60.33%) of the study subjects were rural residents, and more than half 12,653 (62.94%) of mothers do not perceive distance to health facility as big problem. About 12,462 (53.84%) of respondents had low community level media exposure. More than half, 11,886 (51.36%) of mothers of children aged 6–23 months had high community level poverty, and about 12,112 (52.33%) of them had low community-level literacy (**Table 2**).

**Table 2:** Individual and community level characteristics of participants, pooled data from Sub-Saharan African countries DHS from 2019- 2023(weighted n = 23,145).

Variables	Categories	Weighted frequency (n)	Percentage (%)
**Age of child in month**	6-8	4,159	17.97%
	9-11	3,842	16.60%
	12-17	8,129	35.13%
	18-23	7,015	30.30%
**Sex of child**	Male	11,810	51.03%
	Female	11,335	48.97%
**Maternal age in years**	15-24	7,452	32.20%
	25-34	10,809	46.70%
	35-49	4,884	21.10%
**Maternal educational level**	No education	7,001	30.25%
	Primary	7,358	31.79%
	Secondary above	8,786	37.96%
**Marital status of mother**	Married	19,698	85.11%
	Unmarried	3,447	14.89%
**Maternal occupation**	Not working	9,827	42.47%
	Working	13,309	57.53%
**Wealth index**	Poor	10,116	43.71%
	Middle	4,620	19.96%
	Rich	8,409	36.33%
**Sex of household head**	Male	17,317	74.82%
	Female	5,828	25.18%
**Family size**	<=5	9,821	42.43%
	>5	13,324	57.57%
**Number of under-five children**	One	8,610	37.20%
	Greater than one	14,535	62.80
**Age at first birth in years**	<20	12,063	52.12%
	>=20	11,082	47.88%
**Place of delivery**	Home	3,977	17.18%
	Health facility	19,168	82.82%
**Post Natal checkup**	No	15,366	66.39%
	Yes	7,779	33.61%
**Attended ANC visit**	No	1,851	8.00%
	Yes	21,294	92.00%
**Frequency of ANC**	<4 times	8,701	37.59%
	>=4 times	14,444	62.41%
**Mode of delivery**	Vaginal	20,714	89.50%
	Caesarian Section	2,431	10.50%
**Time of 1**^**st**^ **ANC**	0-3 month	12,284	53.07%
	>=4 month	10,861	46.93%
**Preceding birth interval**	<=24 month	8,797	38.01%
	>24 month	14,348	61.99%
**Birth order**	First	5,708	24.66%
	2^nd^ −4^th^	11,847	51.19%
	5^th^ and above	5,590	24.15%
**Twin status**	Single	22,376	96.68%
	Multiple	769	3.32%
**Birth size**	Large	5,631	27.18%
	Average	11,082	53.49%
	Small	4,005	19.33%
**Currently breast feeding**	No	5,213	22.52%
	Yes	17,932	77.48%
**Time for initiation of breast milk**	Immediate	15,753	68.06%
	Late	7,392	31.94%
**Media exposure**	No	6,100	26.36%
	Yes	17,045	73.64%
**Distance to water sources**	<=30’	9,177	39.65%
	>30’	13,968	60.35%
**Distance to health facility**	Big problem	7,449	37.06%
	Not big problem	12,653	62.94%
**Community ANC coverage**	Low	16,093	69.53%
	High	7,052	30.47%
**Place of residence**	Urban	9,183	39.67%
	Rural	13,962	60.33%
**Community media exposure**	Low	12,462	53.84%
	High	10,683	46.16%
**Community poverty**	Low	11,259	48.64%
	High	11,886	51.36%
**Community literacy**	Low	12,112	52.33%
	High	11,033	47.67%

### Pooled prevalence of sugar sweetened beverage or snack consumption

**Fig 2:**
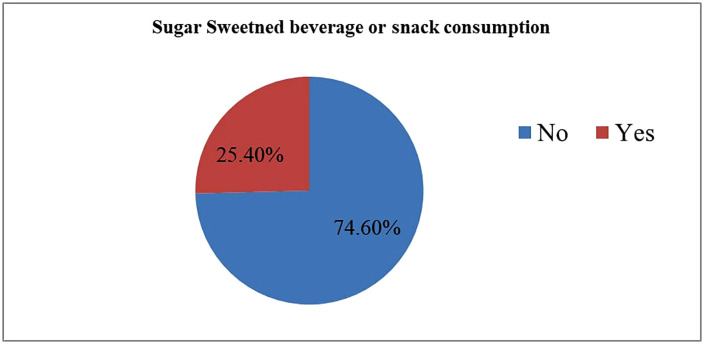
Prevalence of SSB/SN consumption among children aged 6–23 months in SSA (n = 23,145).

In this study, 5,879 (25.40%, 95% CI: 24.84% − 25.96%) infant and young children aged 6–23 months consumed sugar sweetened beverages or snacks during the day preceding the survey (**Fig 2**).

**Fig 3:**
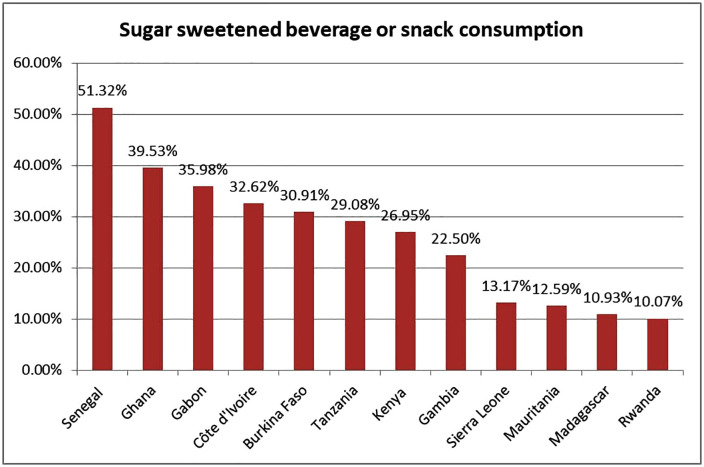
SSB/SN consumption percentages among children 6–23 months across SSA countries (n = 23,145).

The study also showed that sugar sweetened beverage and snack consumption increases with increasing wealth status, with the proportion of infants who consumed SSB/SN being lowest among infants and young children from poor households (17.18%) and highest among rich household families (35.99%). It is also found that the proportion of SSB/SN consumption among infants and young children is higher among urban (34. 46%) residents than rural (19.44%) residents. The consumption of sugar sweetened and snack consumption was high in Senegal (51.32%), followed by Ghana (39.53%), and low in Rwanda (10.07%) (**Fig 3**).

### Measures of variation and model fitness

A significant variance in the chance of consuming SSB/SN was found in the null model (community variance = 0.43, p 0.000). In addition, the ICC value of 0.1147 in the null model indicates that 11.47% of variance in SSB/SN consumption was due to cluster/community variations. Then, with the null model, community-level variables were used to generate Model II. According to the ICC value from Model II, cluster variations were the basis for 7.99% of the differences in SSB/SN consumption. In the final model (model III), which attributed approximately 10.49% of the variation in the likelihood of SSB/SN consumption to both individual and community-level variables. Furthermore, the median odds ratio (MOR) was 1.80 in the final model, which indicates that when children move from a low to high SSB/SN consumption area, the risk of being exposed to SSB/SN consumption increased by 1.80 times. The Proportional Change in Variance (PCV) in the final model of this study was 9.30% indicating that about 9.30% of cluster variation in SSB/SN consumption has been explained by both individual level and community level variables included compared to the null model. The final model (model III), which incorporates both individual and community level variables was the best-fitted model with the smallest Akaike Information Criterion (AIC) and the largest Log-likelihood ratio (LLR) (**Table 3**).

**Table 3:** Model comparison and random effect analysis for sugar sweetened beverage or snack consumption among infant and young children aged 6–23 months in Sub-Saharan African countries, DHS 2019-2023 (*n* = 23,145).

Parameter	Null model	Model I	Model II	Model III*
Variance	0.43	0.60	0.29	0.39
ICC (%)	11.47%	15.41%	7.99%	10.49%
MOR	1.86	2.09	1.66	1.80
PCV	Reference	−0.3953	0.3256	0.0930
**Model Fitness**				
LLR	−12605.68	−9744.42	−12110.03	−9734.56
AIC	25215.37	19510.84	24234.06	19491.12
BIC	25231.55	19598.23	24290.71	19578.52

Key: * best fitted model.

### Factors associated with Sugar sweetened beverage or snack consumption among infant and young children aged 6–23 months

To determine factors associated with SSB/SN consumption, variables with p ≤ 0.25 at bivariable multilevel analysis were entered in to multivariable multi-level regression analysis. Accordingly, in the final fitted model of multivariable multilevel logistic regression, current age of child, current breast feeding status of child, media exposure, wealth index, maternal age at first birth, Post natal checkup, maternal ANC visit, number of under-five children in the household, distance to health facility, place of residence and community level media exposure were independent predictors of SSB/SN consumption among infant and young children aged 6–23 months old at p < 0.05.

The multivariable multilevel analysis revealed that children in age group of 18–23 [AOR = 3.77; 95% CI (3.07, 4.63)], 12–17[AOR = 2.83; 95% CI (2.26, 3.54)], and 9–11 [AOR = 1.95; 95% CI (1.62, 2.35)] month had higher odds to consume SSB/SN compared with children in age group of 6–8 months. Children who were currently being breastfed had 30% lower odds [AOR = 0.70; 95% CI (0.59, 0.82)] of consuming SSB/SN compared to their counterparts.

Those mothers who were exposed to media were 1.59 times [AOR = 1.59; 95% CI (1.28, 1.98)] more likely to give their children SSB/SN. Households wealth status was also another predictor of SSB/SN consumption, in which infant and young children from rich [AOR = 2.31; 95% CI (1.87, 2.85)] and middle [AOR = 1.39; 95% CI (1.11, 1.73)] wealth status were 2.31 and 1.39 times more likely to consume SSB/SN respectively compared to those from poor wealth status. Moreover, children aged 6–23 months whose mothers gave birth for the first time at age 20 years or older were 12% less likely [AOR = 0.88; 95% CI: (0.81, 0.96)] to consume SSB/SN compared to those whose mothers had their first child before age 20. Children in households with more than one under-five child were 11% less likely to consume SSB/SN compared with those in households with only one under-five child [AOR = 0.89; 95% CI (0.80, 0.99)].

This study also found that children of mothers who had PNC checkups were 1.18 times more likely to consume SSB/SN compared with their counterparts [AOR = 1.18; 95% CI (1.05, 1.33)]. Similarly, children aged 6–23 months whose mothers attended ANC had 60% [AOR = 1.60; 95% CI (1.15, 2.22)] higher odds of consuming SSB/SN compared to children whose mothers did not attend ANC. Furthermore, the interpretation of the result showed that children whose mothers do not perceive distance to a health facility as a big problem had 14% lower odds (OR = 0.86) of consuming SSB/SN compared to children whose mothers do perceive distance as a big problem [AOR = 0.86; 95% (0.76, 0.98)].

From community level variable factors, Children from rural areas were 32% less likely to consume SSB/SN compared with those from urban areas [AOR = 0.83; 95%CI (0.68, 1.02)]. Finally, children from communities with high media exposure were 2.22 [AOR = 2.22, 95% CI (0.85, 5.81)] times more likely to consume SSB/SN compared with their counterparts (Table 4).

**Table 4:** Individual and community-level factors associated with SSB/SN consumption among infant and young children aged 6–23 months in 12 Sub-Saharan Africa countries (weighted n = 23,145).

Variables	Category	SSB/SN Consumption		Model I	Model II	Model III
		Yes, n (%)	No, n (%)	AOR (95% CI)	AOR (95% CI)	AOR (95% CI)
Age of child in month	6-8	541(12.98%)	3619(87.02%)			
	9-11	791(20.58%)	3,052(79.42%)	1.95(1.62, 2.35)		1.95 (1.62, 2.35)*
	12- 17	2,210(27.19%)	5,919(72.81%)	2.83(2.26, 3.54)		2.83 (2.26, 3.54)*
	18-23	2,337(33.32%)	4,676(66.68%)	3.76(3.06, 4.63)		3.77(3.07, 4.63)*
Currently breast feeding	No	1,954(37.48%)	3,259(62.52%)			
	Yes	3,924(21.88%)	14,008(78.12%)	0.70(0.60, 0.82)		0.70(0.59, 0.82)*
Initiation of BF after delivery	Immediate	3,627(23.03%)	12,125(76.97%)			
	Late	2,252(30.46%)	5,141(69.54%)	0.98(0.87, 1.11)		0.98 (0.87, 1.11)
Birth size	Large	1,660(29.48%)	3,971(70.52%)			
	Average	3,304(29.81%)	7,778(70.19%)	0.98(0.87, 1.10)		0.98(0.87, 1.10)
	Small	916(22.87%)	3,089(77.13%)	0.89(0.68, 1.15)		0.89(0.68, 1.15)
Birth order	First	1,533(26.86%)	4,175(73.14%)			
	2^nd^ −4^th^	3,204(27.04%)	8,643(72.96%)	1.00(0.87, 1.15)		0.99(0.87, 1.15)
	5^th^ and above	1,141(20.41%)	4,449 (79.59%)	0.90(0.74, 1.00)		0.90(0.74, 1.10)
Age of mother	15-24	1,786(23.97%)	5,666(76.03%)			
	25-34	2,870(26.55%)	7,939 (73.45%)	1.09(0.91, 1.31)		1.09 (0.90, 1.30)
	35-49	1,223(18.35%)	3,661(81.65%)	1.18(0.95, 1.46)		1.17(0.95, 1.44)
Mother education	No education	1,456(20.8%)	5,545(79.20%)			
	Primary	1,584(21.53%)	5,774(78.47%)	1.16(0.91, 1.46)		1.15(0.90, 1.46)
	Secondary & above	2,839(32.31%)	5,947(67.69%)	1.31(0.99, 1.72)		1.29(0.98,1.70)
Maternal occupation	Not working	2,413(24.55%)	7,414(75.45%)			
	Working	3,462(26.01%)	9,847(73.99%)	1.07(0.93, 1.23)		1.08(0.93, 1.24)
Media exposure	No	808.44901	5,291.685			
	Yes	5,070.063	11,974.23	1.60(1.29, 1.97)		1.59 (1.28, 1.98)*
Wealth index	Poor	1,738(17.18%)	8,378(82.82%)			
	Middle	1,114(24.11%)	3,506(75.89%)	1.44(1.13, 1.82)		1.39(1.11, 1.73)*
	Rich	3,027(35.99%)	5,382(64.01%)	2.54(1.94, 3.33)		2.31(1.87, 2.85)*
Age at first birth	< 20 years	2,915(24.16%)	9,148(75.84%)			
	>=20 years	2,964(26.75%)	8,118(73.25%)	0.88(0.80, 0.96)		0.88(0.81, 0.96)*
Family size	<=5	2,643(26.91%)	7,177(73.09%)			
	>5	3,236(24.29%)	10,088(75.71%)	0.98(0.91, 1.05)		0.98(0.91, 1.05)
Number of U-5 children	One	2,488(28.90%)	6,122(71.1%)			
	Greater than one	3,391(23.33%)	11,144(76.67%)	0.89(0.79, 0.99)		0.89 (0.80, 0.99)*
Distance to water source	<=30’	1,879(20.48%)	7,298(79.52%)			
	>30’	3,999.7	9,968.012	1.05 (0.95, 1.16)		1.03(0.93, 1.15)
Place of delivery	Home	523(86.85%)	3,454(13.15%)			
	Health facility	5,356(27.94%)	13,812(72.06%)	1.27 (1.00, 1.63)		1.26 (0.99, 1.59)
PNC	No	3,635(23.66%)	11,731(76.34%)			
	Yes	2,244(28.85%)	5,535(71.15%)	1.18(1.05, 1.32)		1.18(1.05, 1.33)*
Mode of delivery	Vaginal	5,110(24.67%)	15,604(75.33%)			
	CS/yes	768(31.59%)	1,663(68.41%)	0.91(0.73, 1.12)		0.91(0.74, 1.12)
ANC attendance	No	259(13.99%)	1,592(86.01%)			
	Yes	5,619(26.39%)	15,675 (73.61%)	1.59(1.15, 2.21)		1.60(1.15, 2.22)*
Frequency of ANC	<4 times	1,724(19.81%)	6,977(80.19%)			
	>=4 times	4,155(28.77%)	10,289(71.23%)	1.08 (0.94, 1.23)		1.08(0.95, 1.23)
Distance to health facility	Big problem	1,799(24.15%)	5,650(75.85%)			
	Not big problem	3,853(30.45%)	8,800(69.55%)	0.87(0.77, 0.98)		0.86(0.76, 0.98)*
Place of residence	Urban	3,164(34.46%)	6,018(65.54%)			
	Rural	2,715(19.44%)	11,248(80.56%)		0.48(0.33, 0.70)	0.83 (0.68, 0.95)*
Community ANC coverage	Low	3,655(22.71%)	12,438(77.29%)			
	High	2,224(31.54%)	4,828(68.46%)		1.45(0.75, 2.81)	1.10(0.55, 2.24)
Community media exposure	Low	2,378(19.08%)	10,084(80.92%)			
	High	3,500(32.76%)	7,183(67.24%)		1.62(0.69, 3.81)	2.22(1.65, 5.81)*
Community poverty	Low	3,251(28.87%)	8,008(71.13%)			
	High	2,628(22.11%)	9,258(77.89%)		0.88(0.45, 1.73)	0.78(0.36, 1.71)
Community literacy	Low	2,744(22.66%)	9,368(77.34%)			
	High	3,135(28.41%)	7,898(71.59%)		1.14(0.50, 2.60)	0.96(0.39, 2.32)

Key: AOR = Adjusted Odds Ratio, CI = Confidence Interval * significant at p-value< 0.05.

## Discussions

The finding of this study revealed that, the pooled prevalence of SSB/SN consumption among infants and young children aged 6–23 months among 12 SSA countries was 25.40% (95% CI: 24.84% − 25.96%). This figure was lower than the studies employed in the USA (50.5%) [23], Asia (75%), Africa (46%) [24], Ethiopia (49%) [25], Bangladesh (62%) [26], and Nepal (91%) [27]. The prevalence in this study was also higher than the study reported in Cambodia (7%) [28] by 2024. The reason for this discrepancy might be attributed to differences in study area, sample size, study period, and socioeconomic status of study participants. The previous studies were conducted from a single area with a small sample size whereas this study used the pooled most recent data from 12 countries with a large sample size. The difference might also be due to differences in ascertaining the outcome variable.

Multilevel analysis of factors linked to SSB/SN consumption showed that children in older age groups had a higher likelihood of consuming these products compared to younger ones. This finding is consistent with evidence from studies conducted in the USA [22], and Canada [29]. This might be attributed to developmental, environmental, or behavioral factors. As children approach toward two-year, they will get greater autonomy in food choices, are more likely to be exposed to family eating patterns, and may begin to express personal preferences for sweet or processed foods. Furthermore, it might also be due to the transition from complementary feeding to family foods typically occurs during this age, which may explain the sharp rise in SSB/SN or unhealthy food intake.

The findings of this study showed that children who were currently being breastfed were less likely to consume SSB/SN. This is in-line with the study findings conducted in Brazil [30]. It is also in agreement with the WHO recommendations, suggesting that continued breastfeeding during infancy and early childhood supports healthier complementary feeding practices**.** This is because breast milk continues to meet a significant portion of their nutritional and energy requirements during the complementary feeding period, and breastfed children may rely less on energy-dense, less nutrient, or sweet beverages/snacks [31]. This could be, since breastfeeding gives not only optimal nutrition, but also satiety, which might reduce the frequency or desire to feed sweet beverages or snacks as a substitute. More over continued breast feeding may delay the introduction of sugary foods early, which will shape taste preferences or better eating habit [32]. This study also revealed as children from households with rich and middle wealth status were more likely to consume SSB/SN as compared to those from poor wealth status. This is consistent with the study finding reported in West Africa [32], and studies found by Huffman et’al [24]. This might be attributed to the fact that the richest families may have the income to buy sweet beverages and snacks, and the poorest families may lack the income to buy even cheap snack foods. However, a study among young children in urban Nepal noted a negative relationship between wealth status and increased contribution of sweet beverages and snack foods to total energy intake, with the poorest wealth status were more likely of having diets dominated by these products [5]. Such difference might be explained in different context of consumption as though large group of wealthy children may consume such products, poor families might also be depend on highly cheap sugar sweetened beverages or snacks for calories due to the increased cost of healthy foods.

The result in this study suggests that delayed childbearing may have a protective effect against the introduction of SSB/SN in young children’s diets. Accordingly, children aged 6–23 months whose mothers gave birth for the first time at age 20 years or older had 12% lower odds of consuming SSB/SN as compared to those whose mothers had their first child before age 20. This finding aligns with global evidence suggesting that early childbearing age is a risk factor for poor child health outcomes, including malnutrition and increased unhealthy dietary behaviors [33]. This might be explained as women who become mothers at an older age are more likely to have greater exposure to health information which can positively influence health-seeking behaviors and infant feeding practices [34]. In contrast, younger mothers, may lack the same level of health literacy and life experience, which could lead to suboptimal feeding choices, including the introduction of sugar sweetened beverages and sweet snacks which might be used as rewards, or convenient feeding solutions in households [35]. Therefore, delaying the first birth may provide long-term benefits not only for maternal outcomes but also for child health and nutrition.

The presence of more than one under five child in a household was associated with a lower likelihood of SSB/SN consumption among children aged 6–23 months in this study. This is supported by a study conducted by Vidhyashree M. et’al [36], The proposed explanation for this might be families with more than one young child may adopt more structured feeding routines, may have greater dependence on shared meals and home-prepared foods, which are often more traditional and less processed. As a result, the opportunity to offer sweet snacks or beverages might be reduced and avoid costly snack items. In addition, households with multiple children, especially of similar age competition or shared influence might skew toward more balanced or limited snacking patterns. In contrast to the current study finding a study reported by Poti et’al [37] found that households with more children were more likely to consume of ultra-processed foods, linked to convenience and affordability.

Although Antenatal Care (ANC) or post-natal checkup (PNC) visits are opportunities for health professionals to provide essential maternal and child nutrition education and increase IYCF knowledge and practice among mothers [38], this study found that children of mothers who had ANC and PNC visit had higher odds of consuming SSB/SN. The potential explanations for this relationship might suggest that a disconnect between the child feeding advice provided during ANC and postnatal care visits and its actual implementation in the home environment. Additionally, the finding might reflect, a gap in the quality of dietary counseling provided to mothers regarding the introduction of sugar-sweetened snacks or beverages to their infants. The focus of counseling might be simply on general health advice, rather than on specific dietary recommendations for infants. As a result, mothers attending ANC or PNC may not receive adequate guidance on the problems associated with early sugar sweetened beverage and snack consumption [32]. It might also suggest, health providers may not consistently emphasize the risks of introducing sweetened beverages or snacks, or the information provided may not be effectively translated into practice. The other possible explanation might be due to mothers who attend ANC visit or PNC may be part in a higher socioeconomic class, which could lead to greater purchasing power for processed and sugary foods [37]. Moreover, these families might live in urban settings, where access to sweetened beverages or snacks is higher [24]. This finding highlights the need to strengthen nutrition counseling within ANC and PNC services, ensuring that messages include not only general feeding practices but also the health risks of sugar sweetened beverages and foods. Additionally, it suggests that broader strategies, including community-based education, regulation of marketing, for sugary products, and promotion of healthy alternatives may be necessary to reduce childhood consumption of SSB/N.

The current study reported that children whose mothers do not perceive distance to a health facility as a big problem were less likely to consume SSB/SN as compared to children whose mothers do perceive distance as a big problem. This might be due to the fact proximity of health facilities leads to better utilization of maternal or child health services, including child nutrition counseling, and acts as an important platform for educating mothers about healthy child feeding including the risks associated with the introduction of sugary snacks and beverages [39]. In addition mothers who can easily access health facilities may get timely and better nutritional information for reducing the feeding of SSB/SN to their child. Moreover, when distance to health facility is perceived as a barrier, mothers might delay for health services and may miss key opportunities to receive evidence-based information on child feeding. These gaps may be filled by less reliable sources of information commercial advertising, or family traditions, which often promote the use of sweet beverages and snacks [40].

Children whose mothers had media exposure were more likely to consume SSB/SN than children whose mothers had not media exposure. Similarly, children from a community with high media exposure were 2.22 times more likely to consume SSB/SN as compared to their counterparts. Research findings from the UK [41] support this. This might be due to the outweigh effect of advertisements for unhealthy food products, particularly sugar sweetened beverages and snacks, through media than messaging on healthy dietary media. The finding is also consistent with the growing evidence that media exposure can affect infant feeding behaviors, by increasing exposure to advertisement of unhealthy food products, especially sugar sweetened beverages and snacks. This is because there is an evidence that advertisement of sugary foods influences purchasing decisions, including feeding of infants and young children [42]. Moreover, high community media exposure might reflects the presence of mass media that will carry advertising for sugary and processed foods. These advertisements can shape maternal perceptions of what constitutes appropriate foods for their young children, even if such foods are not recommended in infant and young child feeding (IYCF) guidelines. The strong association might also suggest that media influence at the community level reflects a shared environment in which SSB/SN promotion is common and less likely to be counterbalanced by health communication methods. These findings align with existing literature that underscores the role of media in shaping dietary practices, including those related to early childhood feeding. Exposure to advertisements promoting sugar-sweetened beverages (SSBs) and processed snacks has been shown to increase the likelihood of their consumption in children [43].

The current study also showed that children who are rural residents were less likely to consume SSB/SN compared with their counterparts. This finding is consistent with prior research in Bangladesh [44] and Tanzania [45]. A plausible explanation for this result could be that most urban mothers of children came from higher socioeconomic status than their rural counter parts, which may have facilitated their access to SSB/SN through supermarkets and food vendors, and the urban food environment by itself is more likely to have wide range of options for advertisement, to access and to choose less nutritious foods like SSB/SN [46].

### Strengths and limitations of the study

The use of a nationally representative, most recent, large sample size data across tewelve countries in SSA to determine the pooled prevalence of SSB/SN consumption and identify both individual and community-level factors among infant and young children aged 6–23 months is the strength of this study. The use of multilevel analysis advanced statistical model and including 12 countries for more generalizability are also other strengths of this study. Despite these strengths, this study also has some limitations. First, the causal relationship between the dependent variable and the independent variables could not be established due to the cross-sectional nature of the study design. The possibility of recall bias is also likely, since the DHS survey depends on self-reporting. In addition, absence of data on potential comorbidities, intervention or treatment status and lack of adjustment for those potential confounding factors could impact dietary behavior of study subjects.

## Conclusions

In this study, one out of four infants and young children aged 6–23 months consumed sugar sweetened beverages or snacks. Current age of child, current breast feeding status of child, media exposure, wealth index, maternal age at first birth, Post natal checkup, maternal ANC visit, number of under-five children in the household, distance to health facility, place of residence and community level media exposure were factors significantly associated with sugar sweetened beverage or snack consumption among infant and young children aged 6–23 months old.

Thus, efforts should be made to disseminate educational messages on the risks of early introduction of SSBs and snacks during routine maternal and child health contacts, including antenatal care (ANC) and postnatal visits. Nutrition focused counseling should reinforce and strength the importance of breast feeding. Furthermore, special attention should be paid to regulate the marketing of SSB/snacks directed at parents and caregivers. Community-level nutrition education programs should also be done to address the strong effect of community media exposure. Health promotion interventions should target urban and middle- to high-income families to encourage healthy purchasing and feeding practices. Design age-specific interventions and support, particularly for those older age group children and with early maternal age at first birth who may be more likely to introduce SSB/SN to their child might also be needed.

## Abbreviations

AIC: Akaike Information Criterion
ANC: Ante Natal Care
DHS: Demographic and Health Survey
ICC: Intra-class Correlation Coefficient
IYCF: Infant and Young Children Feeding
LMICS: Low and Mid Income Countries
MOR: Median Odds Ratio
PCV: Proportional Change in Variance
PNC: Post Natal Care
SSA: Sub-Saharan Africa
SSB/SN: Sugar Sweetened Beverage or Snack
UNICEF: United Nations International Children’s Emergency Fund
WHO: World Health Organization
